# LXRα Regulates Hepatic ChREBPα Activity and Lipogenesis upon Glucose, but Not Fructose Feeding in Mice

**DOI:** 10.3390/nu9070678

**Published:** 2017-06-29

**Authors:** Qiong Fan, Rikke C. Nørgaard, Christian Bindesbøll, Christin Lucas, Knut Tomas Dalen, Eshrat Babaie, Harri M. Itkonen, Jason Matthews, Hilde I. Nebb, Line M. Grønning-Wang

**Affiliations:** 1 Department of Nutrition, Institute of Basic Medical Sciences, University of Oslo, 0317 Oslo, Norway; r.c.norgaard@medisin.uio.no (R.C.N.); christin.lucas@medisin.uio.no (C.L.); k.t.dalen@medisin.uio.no (K.T.D.); jason.matthews@medisin.uio.no (J.M.); h.i.nebb@medisin.uio.no (H.I.N.); 2 Department of Molecular Medicine, Institute of Basic Medical Sciences, University of Oslo, 0317 Oslo, Norway; christian.bindesboll@medisin.uio.no; 3 Centre for Molecular Medicine Norway, University of Oslo, 0318 Oslo, Norway; eshrat.babaie@ncmm.uio.no; 4 Prostate Cancer Research Group, Centre for Molecular Medicine (Norway), University of Oslo and Oslo University Hospitals, 0318 Oslo, Norway; Harri_Itkonen@hms.harvard.edu; 5 Department of Pharmacology and Toxicology, University of Toronto, Toronto, ON M5S1A8, Canada

**Keywords:** LXR, ChREBP, *de novo* lipogenesis (DNL), *O*-GlcNAc

## Abstract

Liver X receptors (LXRα/β) and carbohydrate response element-binding proteins (ChREBPα/β) are key players in the transcriptional control of hepatic *de novo* lipogenesis. LXRα/β double knockout (LXRα^−/−^/β^−/−^) mice have reduced feeding-induced nuclear *O*-linked *N*-acetylglucosamine (*O*-GlcNAc) signaling, ChREBPα activity, and lipogenic gene expression in livers, suggesting important roles for LXRs in linking hepatic glucose utilization to lipid synthesis. However, the role of LXRs in fructose-induced ChREBP activation and lipogenesis is currently unknown. In this study, we studied the effects of high fructose or high glucose feeding on hepatic carbohydrate metabolism and lipogenic gene expression in livers from fasted (24 h) and fasted-refed (12 h) wild type and LXRα knockout (LXRα^−/−^) mice. Hepatic lipogenic gene expression was reduced in glucose fed, but not fructose fed LXRα^−/−^ mice. This was associated with lower expression of liver pyruvate-kinase (*L-pk*) and *Chrebpβ*, indicating reduced ChREBPα activity in glucose fed, but not fructose fed mice. Interestingly, ChREBP binding to the *L-pk* promoter was increased in fructose fed LXRα^−/−^ mice, concomitant with increased glucose-6-phosphatase (*G6pc*) expression and *O*-GlcNAc modified LXRβ, suggesting a role for LXRβ in regulating ChREBPα activity upon fructose feeding. In conclusion, we propose that LXRα is an important regulator of hepatic lipogenesis and ChREBPα activity upon glucose, but not fructose feeding in mice.

## 1. Introduction

Diets rich in the simple sugars glucose and fructose stimulate hepatic *de novo* lipogenesis (DNL) and increase circulating triglycerides in humans and rodents [1,2,3,4]. Many of the enzymes involved in DNL and triglyceride synthesis are primarily regulated at the transcriptional level in a coordinate manner through multiple transcription factors in response to glucose and insulin [5]. Three transcription factors have been identified as particularly important for regulation of lipogenesis: the liver X receptors (LXRα; Nuclear Receptor Subfamily 1 Group H Member 3 (NR1H3) and LXRβ; Nuclear Receptor Subfamily 1 Group H Member 2 (NR1H2)), sterol regulatory element-binding protein 1c (SREBP-1c), and carbohydrate response element-binding protein-α (ChREBPα) [5].

The LXRs are classically known as oxysterol-activated nuclear transcription factors and members of the nuclear receptor family. LXRs heterodimerize with retinoic X receptor (RXR; Nuclear Receptor Subfamily 2 Group B (NR2B)) family members to regulate the expression of genes involved in cholesterol homeostasis, lipogenesis, glucose metabolism, and inflammation [6]. LXRα is the predominantly expressed isoform in lipogenic tissues such as liver and adipose, whereas LXRβ is ubiquitously expressed [7]. In response to dietary cholesterol, glucose, and insulin, hepatic LXRs, in particular LXRα, activate transcription of the two other lipogenic transcription factors SREBP-1c and ChREBPα, which alone or in concert with LXRs induce expression of glycolytic and lipogenic enzymes in hepatic DNL, such as glucokinase (*Gk*), liver pyruvate kinase (*L-pk*), ATP citrate lyase (*Acl*), acetyl-CoA carboxylase (*Acc*), fatty acid synthase (*Fasn*), and stearoyl-CoA desaturase-1 (*Scd1*) [6,8]. In addition, LXRs improve glucose tolerance by negatively regulating hepatic glucose-6-phosphate (*G6pc*) expression and glucose production [9,10,11].

We and others have shown that LXRs and ChREBPα are post-translationally modified by *O*-linked *N*-acetylglucosamine (*O*-GlcNAc) in response to high glucose by *O*-GlcNAc transferase (OGT), which increases their lipogenic potential [12,13]. OGT uses UDP-*N*-acetylglucosamine (UDP-GlcNAc), the high energy product of the hexosamine biosynthetic pathway (HBP), as a substrate for reversible *O*-linked GlcNAcylation of nuclear, cytoplasmic, and mitochondrial target proteins affecting transcription, metabolism, apoptosis, organelle biogenesis, and transport [14]. The HBP is a branch of the glycolytic pathway that couples nutrient sensing to cellular metabolism and signaling via activation of OGT [15]. *O*-GlcNAcylation regulates transcription through OGT-associated chromatin-modifying complexes [16]. In this way, glucose not only serves as an energy source and substrate for lipogenesis, but also acts as a signaling molecule in the regulation of glycolytic and lipogenic gene expression [17]. In line with this notion, *O*-GlcNAc signaling is associated with increased ChREBPα activity, enhanced glycogenic and lipogenic gene expression, and hepatic steatosis [13]. We recently reported that insulin-independent glucose-*O*-GlcNAc signaling potentiates LXR-mediated transactivation of the SREBP-1c and ChREBPα promoters, linking glucose metabolism to LXR activation and lipogenesis [12,18]. Furthermore, we reported that LXRs are important for nuclear *O*-GlcNAc signaling, ChREBPα *O*-GlcNAcylation and *L-pk* promoter binding activity, and glycogenic and lipogenic gene expression, including expression of the newly discovered *Chrebpβ* isoform in mouse livers. Collectively, these data suggest that LXRs connect hepatic glucose utilization to lipogenesis via regulation of nuclear OGT and ChREBPα activity [18,19]. However, the specific roles of LXRα and LXRβ in this process are currently unknown.

ChREBPβ is derived from an alternative promoter within exon 1b of the ChREBP gene, resulting in a shorter constitutively nuclear protein lacking most of the low glucose inhibitory domain (LID) in *N*-terminus [20]. *Chrebpβ* expression is low during prolonged fasting and strongly induced following high carbohydrate refeeding in mice [21]. ChREBPα mediates this response in a tissue-specific manner via transactivation of carbohydrate response elements (ChoREs) upstream of and in exon 1b [21]. Interestingly, ChREBPβ conferred a higher transcriptional activity than ChREBPα under both low and high glucose conditions and appears to be the major regulator of lipogenesis in response to dietary carbohydrates [20,22]. Recently, a role for ChREBP and in particular ChREBPβ, in fructose-induced *de novo* lipogenesis was suggested [21,23]. ChREBP null mice are intolerant to high fructose diet, in part by blunted gene expression of fructose-metabolizing enzyme genes, suggesting also a crucial role for ChREBP in fructose metabolism [24]. Interestingly, a recent study showed that ChREBP induces hepatic *G6pc* expression and glucose production by short-term fructose feeding in mice [25], suggesting a role for ChREBP in contributing to selective hepatic insulin resistance.

The objectives of the present study were to investigate the LXRα dependent effect of dietary fructose and glucose on hepatic ChREBPα activity, glycogenic and lipogenic gene expression, intermediate carbohydrate metabolism, and *O*-GlcNAc levels by using wild type and LXRα deficient mice. The results from this study indicate that LXRα is important in hepatic DNL and ChREBPα activity upon glucose, but not fructose feeding in mice.

## 2. Materials and Methods

### 2.1. Materials

Formaldehyde (F1635) and UDP-GlcNAc (U4375) were purchased from Sigma Aldrich (St. Louis, MO, USA). UltraPure™ Phenol: Chloroform:Isoamyl Alcohol (15593-031) was from Invitrogen Aldrich (Thermo Fisher Scientific, Waltham, MA, USA). All other chemicals were of the highest quality available from commercial vendors.

### 2.2. Animals and Treatment

Wildtype (LXRα^+/+^) and LXRα deficient (LXRα^−/−^) mice were housed in a temperature-controlled (22 °C) facility with a strict 12 h light/dark cycle. Mice had free access to standard chow diet (SDS diets, RM3, #801190, consisting of 12% calories from fat, 27% from protein, and 61% from carbohydrate) and water at all times prior to experiments. The generation of the LXRα^−/−^ mice has been described previously [26]. The LXRα^+/+^ and LXRα^−/−^ mice used were of mixed genetic background (129/Sv/C57BL/6) backcrossed into the C57BL/6N strain for six generations. Twelve-week-old male mice (*n* = 5) were fasted for 24 h or fasted for 24 h and subsequently refed for 12 h on an isocaloric diet (3.99 kcal/g) containing 60.8% calories from fructose (5BN7) or glucose (5BN8) (TestDiet), 22.6% fat, and 16.7% protein. The mice were sacrificed in a mixed order between fasted and refed groups by cervical dislocation at 7–9 a.m., and tissues were weighed and snap frozen in liquid nitrogen and stored at −80 °C until further analysis. All use of animals was registered and approved by the local veterinary and the Norwegian Animal Research authority (FOTS #5457 and #6378).

### 2.3. Blood Chemistries

Plasma was separated from blood by centrifugation. Plasma insulin was measured using the Ultrasensitive Insulin Kit from Mercodia (Mercodia AB, Uppsala, Sweden) according to the manufacturer’s instructions. Plasma triglycerides (TGs) were determined with a Triglycerides Enzymatic PAP 150 kit (TGPAP 150; BioMérieux, Marcy-l’Étoile, France).

### 2.4. Metabolomics

Liver tissues (*n* = 5 mice for each group) were sent to Metabolon, Inc. (Research Triangle Park, Durham, NC, USA) for metabolomics analysis as described [27]. The metabolomics data is included in Supplementary Table S1.

### 2.5. RNA Extraction, cDNA Synthesis and Real-Time Quantitative PCR (RT-qPCR)

RNA was isolated by phenol chloroform extraction followed by high salt precipitation (0.8 M sodium acetate, 1.5 M NaCl) to avoid contaminating polysaccharides to co-precipitate with RNA. Extracted RNA was further purified using RNeasy spin columns (#74104; QIAGEN, Hilden, Germany). Isolated RNA (500 ng) was reverse transcribed into cDNA using SuperScript III Reverse Transcriptase (Invitrogen, Thermo Fisher Scientific, Waltham, MA, USA) and random hexamer primers. qPCR was performed with 1 µL of the cDNA synthesis reaction using Kapa SYBR FAST qPCR Master Mix (KapaBiosystems, Roche, Basel, Switzerland) on a Bio-Rad CFX96 Touch™ Real-Time PCR Detection System. Gene expression was normalized against the expression of TATA-binding protein (*Tbp*). Assay primers were designed with Primer-BLAST software (NCBI, Bethesda, MD, USA) [28]. Sequences are listed in Supplementary Table S1.

### 2.6. Liver Extracts and Immunoblot Analysis

Nuclear and cytoplasmic proteins were prepared using NE-PER^TM^ Nuclear and Cytoplasmic Extraction kit (Pierce Biotechnology, Thermo Fisher Scientific, Waltham, MA, USA), with the following inhibitors added to the buffers: 1 mM NaF, 1 mM Na_3_VO_4_, 1 mM β-glycerophosphate, 1 μM *O*-GlcNAcase inhibitor GlcNAc-thiazoline, and Complete^TM^ protease inhibitors (Roche Applied Science, Penzberg, Germany). Proteins were separated by Sodium Dodecyl Sulfate Polyacrylamide Gel Electrophoresis (SDS-PAGE) (Bio-Rad, Hercules, CA, USA) and blotted onto Polyvinylidene fluoride (PVDF) membrane (Merck Millipore, Billerica, MA, USA). Primary antibodies used were rabbit anti-mouse LXR (1:500) [19], ChREBP (1:1000; #NB400-135; Novus Biologicals, Littleton, CO, USA), FASN (1:500; #sc-55580; Santa Cruz Biotechnology, Dallas, TX, USA), SCD1 (1:2000; #sc-14719; Santa Cruz Biotechnology, Dallas, TX, USA), α-Tubulin (1:20,000; #T5168; Sigma-Aldrich, St. Louis, MO, USA), Lamin A (1:1000; #L1293; Sigma-Aldrich, St. Louis, MO, USA), OGT (1:1000; #AL25) [29], RL2 (1:1000, #MA1-072, Invitrogen, Thermo Fisher Scientific, Waltham, MA, USA), SREBP-1 (1:1000) [30], and L-PK (1:2000; #MABS148; Merck Millipore, Billerica, MA, USA). Secondary horseradish peroxidase-conjugated anti-mouse (#115-035-174) and anti-rabbit (#211-032-171; Jackson ImmunoResearch Laboratories, West Grove, PA, USA); anti-goat (#605-4302; Rockland, Limerick, PA, USA) antibodies were used at 1:10,000 dilutions. Anti-mouse IgM (#A8786; Sigma-Aldrich, St. Louis, MO, USA) was used at 1:5000 dilutions. Blots were quantified from five mice for each experimental group, using the ImageJ software (NIH, Bethesda, Maryland). All lanes were normalized to loading controls as indicated in figure text (α-tubulin or Lamin A).

### 2.7. Chromatin Immunoprecipitation (ChIP)

ChIP experiments were performed as described previously with modest changes [19]. Briefly, liver tissue was homogenized and crosslinked with 1% formaldehyde/Phosphate-buffered saline (PBS) for 10 min at room temperature. Crosslinking was stopped by 3 min incubation with 125 mM glycine. Samples were washed twice in cold PBS and resuspended in lysis buffer (0.1% SDS, 1% Triton X-100, 0.15 M NaCl, 1 mM Ethylenediaminetetraacetic acid (EDTA), and 20 mM Tris (pH 8.0). Lysed tissue was sonicated to an average size of 200–500 bp fragments using a bioruptor (Diagenode, Seraing, Belgium). Chromatin was immunoprecipitated with 2 µg antibody against ChREBP (NB400-135; Novus Biologicals, Littleton, CO, USA) or rabbit IgG (011-000-002; Jackson ImmunoResearch Laboratory, West Grove, PA, USA) over night at 4 °C. Dynabeads Protein A (Invitrogen) were washed four times in lysis buffer, and then added to the chromatin and rotated at 4 °C for 2 h. Dynabeads were then washed three times with wash buffer 1 (0.1% SDS, 1% Triton X-100, 0.15 M NaCl, 1 mM EDTA, 20 mM Tris (pH 8)); followed by washing once in wash buffer 2 (0.1% SDS, 1% Triton X-100, 0.5 M NaCl, 1 mM EDTA, 20 mM Tris (pH 8)), and then once in wash buffer 3 (0.25 M LiCl, 1% NaDOC, 1% NP-40, 1 mM EDTA, 20 mM Tris (pH 8)), and then once in wash buffer 1. All washing steps were done for five minutes at room temperature. DNA-protein complexes were eluted with 1% SDS and reverse cross-linked overnight at 65 °C. DNA was purified by using the QIAquick PCR Purification Kit (#28104; QIAGEN, Hilden, Germany). DNA enrichment was quantified by quantitative RT-PCR. The binding of ChREBP to the carbohydrate response element (ChoRE) in the promoter-proximal enhancer of *L-pk* has been described previously [13] and the ChIP primers used were as follows: *L-pk* ChoRE (5′-GTCCCACACTTTGGAAGCAT, 5′-CCCAACACTGATTCTACCC). The negative control primers located 2.2 kp downstream from the ChoRE were as follows: 5′-TGCAACTGGGGAACTAGCCA, 5′-AGCTTTGTGTGATGGCTGAAG.

### 2.8. Wheat Germ Agglutinin (WGA) Pulldown

Nuclear extracts (100 µg) were incubated with protein A/G-agarose beads (sc-2003; Santa Cruz Biotechnology) for 1 h at 4 °C. Cleared extracts were transferred to new tubes and incubated with 30 μL of WGA-agarose (Vector Lab, Burlingame, CA, USA) overnight at 4 °C. After four washes (PBS, 0.2% NP-40), proteins were eluted from the beads in 2× Laemmli buffer and separated by SDS-PAGE. The captured proteins were analyzed by immunoblotting.

### 2.9. UDP-GlcNAc Measurements

UDP-GlcNAc in liver tissue was extracted and analyzed using described methods [31] with minor changes. Liver tissue (20 μg) was homogenized in liquid nitrogen-chilled CryoGrinder (OPS Diagnostics, Lebanon, NJ, USA), and resuspended in 0.15 mL cold PBS. Ice-cold ethanol (0.45 mL) was added to the samples, followed by sonication in Diagenode Bioruptor to lyse the cells. The lysate was centrifuged at 16,000× *g* for 10 min at 4 °C, and a third of the supernatant was used for determination of protein concentration using the bicinchoninic acid (BCA) assay kit (Pierce, Thermo Fisher Scientific, Waltham, MA, USA), and the rest was vacuum dried in a Savant DNA100 SpeedVac to measure levels of nucleotide sugars with ion-pair reversed phase High-performance liquid chromatography (HPLC). The preparatory columns for HPLC were done as previously reported [32], while the run-time in HPLC was extended up to 2 h. A standard UDP-GlcNAc (100 μM) was spiked into samples to verify the accurate peak.

### 2.10. Statistical Analysis

Statistical analyses were performed using GraphPad Prims 6 (GraphPad Software Inc., San Diego, CA, USA). All data were presented as means and standard error of the mean (SEM), and error bars for all results were derived from biological replicates rather than technical replicates. Statistical differences between groups were determined by two-way analysis of variance (ANOVA) followed by Tukey’s multiple comparison tests. For all statistical tests *p* < 0.05 was considered statistically significant. For metabolomics data, statistical differences between groups were determined by repeated measures two-way ANOVA.

## 3. Results

### 3.1. Regulation of Genes Involved in Carbohydrate Metabolism by Dietary Fructose and Glucose-Role of LXRα

**Figure 1:**
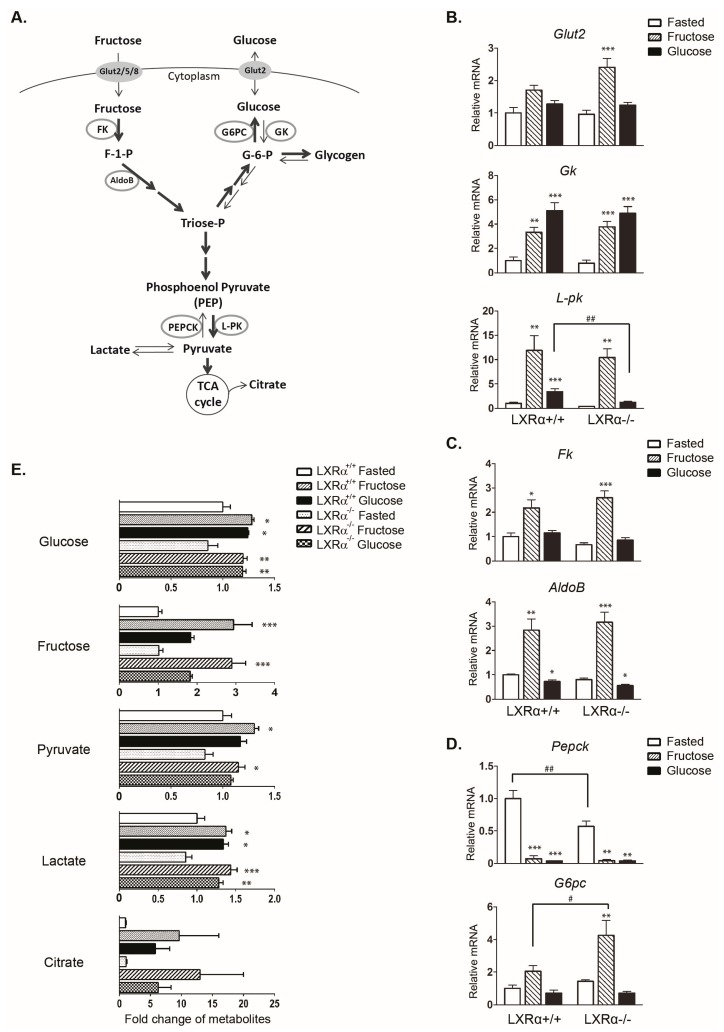
Hepatic liver pyruvate-kinase (*L-pk*) expression by dietary glucose is reduced in LXRα knockout (LXRα^−/−^) mice. The male mice of inbred strain C57 Black 6 (C57BL/6) were fasted 24 h (white bars) or fasted-refed for 12 h on a 60% fructose diet (gray bars) or 60% glucose diet (black bars) as described in Materials and Methods. (**A**) Simplified schematic overview of the genes involved in glycolysis, fructolysis, and gluconeogenesis. F-1-P: fructose-1-phosphate; G-1-P: glucose-1-phosphate; Triose-P: triose phosphate. TCA: tricarboxylic acid; (**B**) Hepatic gene expression of the glycolytic genes glucose transporter 2 (*Glut2*), glucokinase (*Gk*) and *L-pk*; (**C**) Hepatic gene expression of the fructolytic genes fructokinase (*Fk*) and aldolase B (*AldoB*); (**D**) Hepatic gene expression of the gluconeogenic genes phosphoenolpyruvate carboxykinase (*Pepck*) and glucose-6-phosphatase (*G6pc*). Expression of above genes (B-D) were analyzed by real-time quantitative PCR (RT-qPCR) and normalized to TATA-binding protein (*Tbp*); (**E**) Relative metabolite expression levels of hepatic glucose, fructose, pyruvate, lactate, and citrate in response to fructose and glucose feeding in wild type and LXRα^−/−^ mice. Data represent the mean ± standard error of the mean (SEM) (*n* = 5). Significant differences were found using two-way analysis of variance (ANOVA) followed by Tukey’s multiple comparison test (fasted vs. fructose fed and fasted vs. glucose fed were analyzed separately). * *p* < 0.05, ** *p* < 0.01, *** *p* < 0.001 compared to fasted. ^#^ *p* < 0.05, ^##^ *p* < 0.01, compared to LXRα^+/+^ mice.

To examine LXRα-dependent regulation of genes encoding for glucose and fructose metabolizing enzymes in response to fructose and glucose feeding, wild type and LXRα*^−^*^/*−*^ mice were divided into three groups: fasted (24 h), fasted-refed with 60% fructose, or fasted-refed with 60% glucose for 12 h. No statistically significant differences in food intake or body weight were found among the fructose and glucose fed wild type and LXRα*^−^*^/*−*^ mice (Table 1). A schematic representation of the enzymes involved in carbohydrate metabolism (glycolysis, fructolysis, and gluconeogenesis) is presented in Figure 1A. The majority of the analyzed genes were similarly regulated in wild type and LXRα*^−^*^/*−*^ mice. Both diets upregulated glycolytic *Gk* mRNA to a similar degree, and fructose feeding upregulated the ChREBP target gene *L-pk* mRNA more strongly than glucose feeding, which is in agreement with previous observations [33,34] (Figure 1B). Interestingly, glucose-, but not fructose-mediated induction of *L-pk* was significantly attenuated in LXRα*^−^*^/*−*^ mice. Figure 1C shows genes in the fructolysis pathway, fructokinase (*Fk*) and aldolase B (*AldoB*). Only fructose feeding upregulated Fk and AldoB, as previously reported [33]. We observed increased expression of gluconeogenic genes *G6pc* and glucose transporter 2 (*Glut2*) with fructose feeding in the LXRα*^−^*^/*−*^ mice (Figure 1B,D). These observations were concomitant with an approximately 2-fold increase in plasma insulin levels in fructose fed LXRα*^−^*^/*−*^ mice compared to wild type mice on the same diet (Table 1). These data suggest increased glucose production and glucose output in fructose fed mice lacking LXRα.

Metabolite analysis showed significantly increased hepatic levels of glucose and also increased hepatic fructose levels in high glucose fed mice (Figure 1E), suggesting increased activity of aldose reductase upon high glucose consumption, as previously reported [35]. Hepatic glucose levels were also higher in fructose fed mice, in agreement with increased glycogen synthesis and turnover [36,37]. We observed elevated hepatic levels of glycogenic metabolites upon both fructose and glucose feeding, likely derived from the continuous production and turnover of glycogen (Supplementary Table S2). Hepatic pyruvate, lactate, and citrate levels were increased in wild type and LXRα*^−^*^/*−*^ mice upon fructose and glucose feeding (Figure 1E), which correspond with increased carbohydrate oxidation after digestion of these carbohydrate rich diets.

### 3.2. Expression of Hepatic de novo Lipogenic Genes Mediated by Dietary Glucose Is Reduced in LXRα^−/−^ Compared to Wild Type Mice

**Figure 2:**
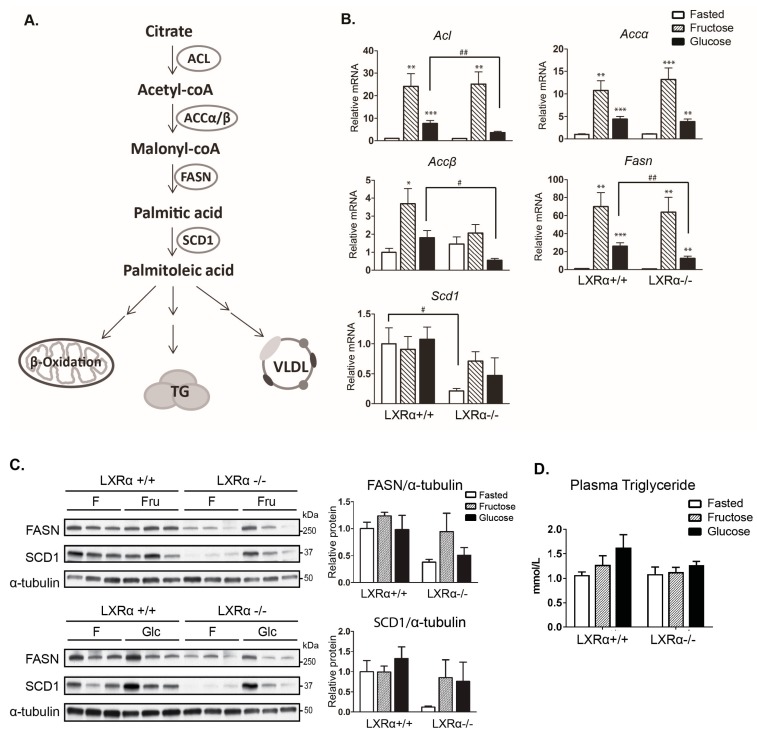
Hepatic *de novo* lipogenic gene expression is dependent on LXRα in response to dietary glucose. (**A**) Simplified schematic overview of genes involved in hepatic *de novo* lipogenesis. VLDL: very low-density lipoprotein; (**B**) Hepatic gene expression of lipogenic genes ATP citrate lyase (*Acl*), acetyl-CoA carboxylase (*Acc*), fatty acid synthase (*Fasn*), and stearoyl-CoA desaturase-1 (*Scd1*) were analyzed by quantitative RT-PCR and normalized to *Tbp*; (**C**) Cytosolic lysates were immunoblotted with antibodies against FASN and SCD1 with α-Tubulin as loading control. Each lane represents independent mice from each experimental group. One representative western blot is shown (*n* = 3). Quantification of cytosolic FASN and SCD1 proteins was analyzed by ImageJ (*n* = 5). F: fasted mice; Fru: fructose-fed mice; Glc: glucose-fed mice; (**D**) Plasma triglycerides (TG). Data represent the mean ± SEM (*n* = 5). Significant differences were found using two-way ANOVA followed by Tukey’s multiple comparison test (fasted vs. fructose fed and fasted vs. glucose fed RNA data were analyzed separately). * *p* < 0.05, ** *p* < 0.01, *** *p* < 0.001 compared to fasted. ^#^ *p* < 0.05, ^##^ *p* < 0.01, compared to LXRα^+/+^ mice.

We next addressed the gene expression of central lipogenic enzymes in fasted and refed mice. A schematic representation of DNL and the enzymes involved is presented in Figure 2A. All lipogenic genes assessed, except for *Scd1*, were more strongly upregulated by fructose compared to glucose feeding (Figure 2B), in agreement with previous reports [21,38,39]. Glucose-induced lipogenic gene expression was reduced in LXRα*^−^*^/*−*^ mice (Figure 2B). In line with previous observations in our laboratory [19], *Scd1* expression was not upregulated after high sugar feeding. SCD1 mRNA and protein expression were almost completely abolished in LXRα*^−^*^/*−*^ fasted mice (Figure 2B,C), consistent with a previous study reporting that LXR directly regulate SCD1 expression [40]. Although lipogenic gene expression was significantly induced by both diets in wild type mice, plasma triglyceride levels were not significantly elevated after 12 h refeeding (Figure 2D).

### 3.3. Regulation of Srebp-1 and Chrebpβ Expression Mediated by Dietary Glucose Is Dependent of LXRα

**Figure 3:**
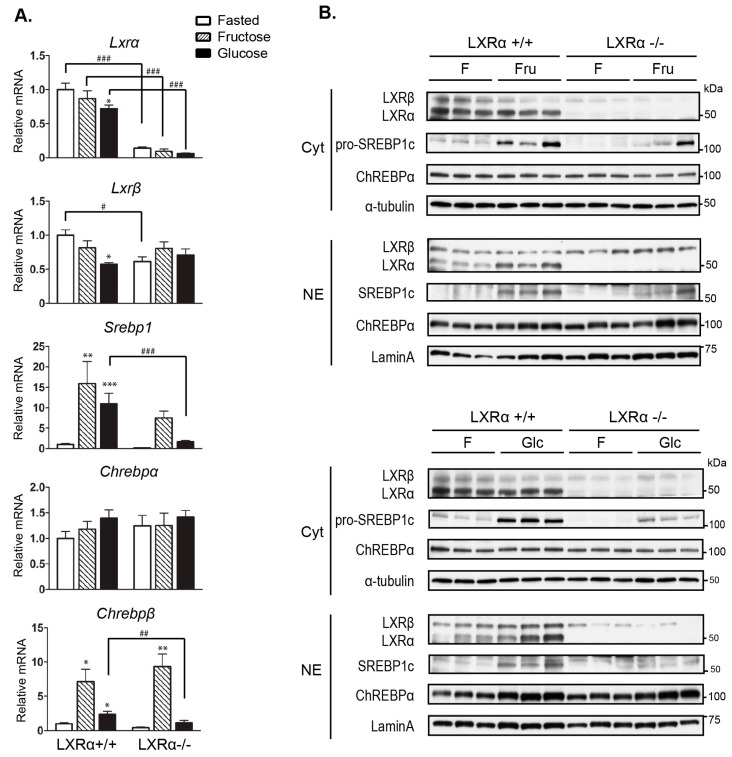
Induction of hepatic *Srebp-1* and carbohydrate response element-binding protein (*Chrebp*)*β* expression by dietary glucose is reduced in LXRα^−/−^ mice. (**A**) Hepatic gene expression of *Lxrα/β*, *Srebp-1*, and *Chrebpα/β* was analyzed by quantitative RT-PCR and normalized to *Tbp*; (**B**) Cytosolic and nuclear lysates were immunoblotted with antibodies against LXR, SREBP-1, and ChREBP with α-Tubulin and Lamin A as loading controls. Each lane represents independent mice from each group. One representative western blot is shown (*n* = 3). Data represent the mean ± SEM (*n* = 5). Significant differences were found using two-way ANOVA followed by Tukey’s multiple comparison test (fasted vs. fructose fed and fasted vs. glucose fed RNA data were analyzed separately). * *p* < 0.05, ** *p* < 0.01, *** *p* < 0.001 compared to fasted. ^#^ *p* < 0.05, ^##^ *p* < 0.01, ^###^ *p* < 0.001 compared to LXRα^+/+^ mice.

LXRs, and particularly LXRα, are known as upstream regulators of SREBP-1c and CHREBPα expression in response to glucose and insulin, and all three transcription factors are involved in the regulation of hepatic lipogenic enzyme genes described above [6,19]. Recent investigations have shown that ChREBPα is activated by dietary fructose, which in turn induces the expression of *Chrebpβ* [21,23]. We did not observe significant changes in *Chrebpα* mRNA levels by both diets in wild type mice, while high fructose feeding more potently induced *Chrebpβ* mRNA expression as compared to glucose (Figure 3A). Compared to wild type mice, *Chrebpβ* mRNA expression was significantly reduced in glucose fed, but not in fructose fed LXRα*^−^*^/*−*^ mice, concomitant with reduced nuclear LXRβ protein expression in glucose-fed LXRα*^−^*^/*−*^ mice (Figure 3B, quantification is shown in Supplementary Figure S1B). This supports our previous observations that both LXR isoforms are necessary to maintain hepatic ChREBPα activity in response to glucose [19], and suggests that LXRβ is able to compensate for the lack of LXRα in regulation of ChREBPα in fructose fed mice. SREBP-1 mRNA and protein (proform) expression were significantly reduced in glucose fed LXRα*^−^*^/*−*^ mice (Figure 3A,B, quantification in Supplementary Figure S1A), supporting the role of LXRα as a central regulator of hepatic *Srebp-1*expression. Notably, we could only detect the ChREBPα protein but not ChREBPβ in our in vivo liver samples, suggestive of a high turnover (rapid degradation) of the constitutively active nuclear ChREBPβ protein.

### 3.4. Dietary Fructose Induces Nuclear O-GlcNAc Signaling

**Figure 4:**
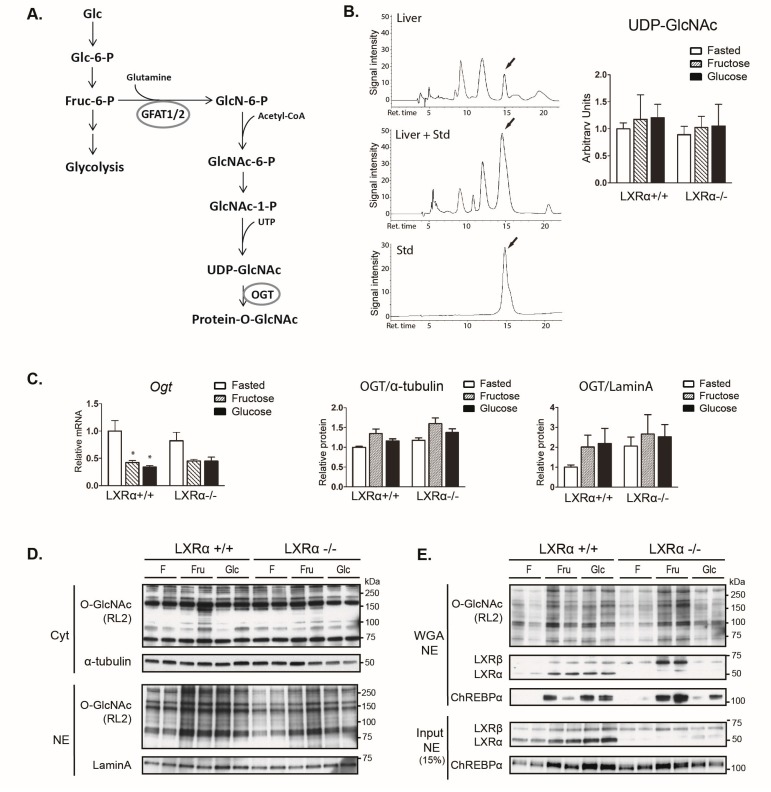

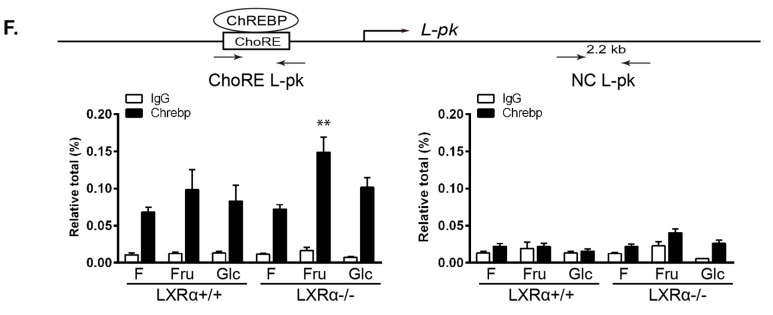
Dietary fructose induces nuclear *O*-GlcNAc signaling. (**A**) Simplified schematic overview of the hexosamine signaling pathway including the rate limiting enzyme GFAT and *O*-GlcNAc transferase (OGT); (**B**) High-performance liquid chromatography (HPLC) analysis of hepatic UDP-GlcNAc levels. Left panel: Example of a typical running profile with or without injection of UDP-GlcNAc standard (Std.) is shown. Right panel: Quantification of the HPLC data normalized to protein concentration; (**C**) Hepatic expression of the *Ogt* gene and cytosolic and nuclear OGT protein were analyzed by quantitative RT-PCR and western blotting/Image J and normalized to *Tbp*, α-tubulin, and Lamin A, respectively. Data represent the mean ± SEM (*n* = 5); (**D**) Cytosolic and nuclear lysates were immunoblotted with anti-*O*-GlcNAc antibody (RL2) with α-Tubulin and Lamin A as loading controls. Each lane represents independent mice from each group. One representative western blot is shown (*n* = 2); (**E**) Nuclear lysates were subjected to wheat germ agglutinin (WGA) beads to precipitate *O*-GlcNAcylated proteins. WGA enriched samples (upper panel) and input lysates (bottom panel) were immunoblotted with antibodies detecting *O*-GlcNAcylated proteins (RL2), LXR and ChREBP. Each lane represents independent mice from experimental groups. Representative western blots are shown (*n* = 2); (**F**) ChREBP binding to the carbohydrate response element (ChoRE) containing region of the *L-pk* promoter and negative control sequence (NC) 2216–2288 bp into the *L-pk* gene after ChoRE sequence was detected by chromatin immunoprecipitation (ChIP) using antibodies against ChREBP or IgG as a control. Data represent the mean ± SEM (*n* = 5). Significant differences were found using two-way ANOVA followed by Tukey’s multiple comparison test. ** *p* < 0.01 compared to fasted.

*O*-GlcNAcylation of ChREBPα has been shown to potentiate its activity, stability, and binding to the *L-pk* promoter in response to refeeding in normal mice and more so in hyperglycemic diabetic mice [13]. The hexosamine biosynthetic pathway (HBP) involving activation of OGT is depicted in Figure 4A. Metabolomics analysis revealed lower levels of *N*-acetylglucosamine-1-P (GlcNAc-1-P) in refed mice with both diets compared to fasting (Supplementary Table S2), suggesting high glutamine-fructose-6-phosphate amidotransferase 2 (GFAT2) activity during fasting, possibly via glucagon-PKA-mediated activation [41]. Although HPLC analysis showed no significant increase in total liver UDP-GlcNAc levels in refed compared to fasted mice (Figure 4B), the fraction of UDP-GlcNAc that is utilized for *O*-GlcNAc modification may be increased towards the nuclear pool after refeeding. Ogt mRNA levels were strongly downregulated upon refeeding (Figure 4C), which is suggestive of a negative feedback regulation due to high OGT activity, as reported previously [42]. We observed increased nuclear protein *O*-GlcNAcylation levels upon fructose refeeding in wild type mice (Figure 4D, quantification in Supplementary Figure S2B). Notably, the *O*-GlcNAcylation of LXRβ was significantly increased in fructose-fed LXRα*^−^*^/*−*^ mice (representative western blots in Figure 4E, quantification (*n* = 4) in Supplementary Figure S2C).

Because ChREBP binding to its cognate DNA-binding site in the *L-pk* promoter is affected by high glucose feeding, *O*-GlcNAc and LXRα/β signaling in rat and mice livers [13,19,43], we next investigated ChREBP recruitment to the *L-pk* promoter in chromatin immunoprecipitation (ChIP) assays. We did not observe a significant increase in ChREBP recruitment after refeeding in wild type mice, but observed a significant increase after fructose feeding in LXRα*^−^*^/*−*^ mice (Figure 4F). This observation, together with increased *O*-GlcNAcylation of LXRβ, suggests that LXRβ may compensate for the lack of LXRα in regulating ChREBPα activity upon fructose feeding.

## 4. Discussion and Conclusions

The results of this study show that LXRα is important for ChREBPα activity upon glucose, but not fructose feeding in the livers of mice, as visualized by lower expression of ChREBPα specific target genes *L-pk* and *Chrebpβ* in LXRα^−/−^ compared to wild type mice. LXRα is also important for the transcriptional control of classical hepatic DNL genes upon glucose feeding, including *Fasn* and *Accβ*. This suggests that LXRα is important in integrating nutritional cues upon glucose feeding upstream of ChREBPα.

In the human diet, fructose and glucose are rarely consumed in isolation. Studies comparing more commonly consumed sucrose and high fructose corn syrup have yielded different results compared to studies with pure fructose or glucose [37]. However, long-term feeding studies (12 h or more) in rat and mice have shown similar fold induction of hepatic lipogenic genes by high fructose compared to sucrose and always above levels in mice fed high glucose [21,33,34,38]. This is likely due to most of the dietary fructose being taken up by the liver whereas only 30% of dietary glucose is metabolized by the liver; the remaining glucose is metabolized in muscle, adipose tissues, brain, kidney, and red blood cells [44]. However, approximately 30–50% of fructose taken up by the liver is converted to glucose and 15% to lactate in part as a fuel source for extrahepatic tissues [37,45]. This suggests additive effects of hepatic glucose and fructose metabolism and signaling in the control of DNL in response to dietary fructose, at least in the late refed phase. This is supported by a study by Matsuzaka et al. [34] that showed the expression of SREBP-1 and FASN above glucose-induced levels after 9 h fructose feeding concomitant with increased plasma glucose and insulin levels. At 6 h refeeding, however, glucose, but not fructose, strongly induced SREBP-1 and FASN expression. Stamatikos et al. reported increased fructose-induced *Accα* and *Fasn* gene expression following inhibition of Fructose-1,6-biphosphatase (FBPase) in human hepatocyte carcinoma (HepG2) cells, suggesting that dietary fructose induces DNL gene expression independently of its ability to generate glucose [21]. However, these results were not verified in primary hepatocytes, suggesting that this effect may be specific for hepatoma cells.

In the present study, we observed similar hepatic glucose levels in fructose and glucose fed mice, but significantly higher pyruvate levels after fructose feeding. This observation is concomitant with more strongly induced expression of the ChREBP target gene *L-pk* upon fructose feeding than glucose feeding, in agreement with previous observations [34]. This supports the notion that fructose activates ChREBP and *L-pk* expression to a higher degree than glucose, thus generating more substrate to DNL [39,46,47]. Notably, *L-pk* expression was almost back to fasting levels in LXRα depleted glucose fed mice, but not in fructose fed mice, suggesting differential roles of LXRα and ChREBP in response to early phase of glucose and fructose metabolism.

We have recently shown that LXRα^−/−^/β^−/−^ mice have reduced nuclear *O*-GlcNAc levels, ChREBPα activity, and lipogenic gene expression in livers as compared wild type mice upon refeeding with a chow diet [19]. Herein, we provide evidence that LXRα is important for ChREBPα activity upon glucose feeding. The effects on hepatic DNL is less striking when depleting LXRα alone, and LXRα was dispensable in fructose fed, but not glucose fed mice, which were also low in LXRβ protein levels. Levels of nuclear LXRβ protein were maintained in fructose fed but surprisingly strongly reduced in glucose fed LXRα^−/−^ mice, suggesting compensation by LXRβ in regulating SREBP-1c and lipogenic gene expression in fructose fed mice. This may be explained, at least in part, by redundancy between the LXRs [48]. Notably, increased WGA-recovery of LXRβ and ChREBPα, which is indicative of increased *O*-GlcNAc modifications of these proteins, was observed in fructose fed LXRα^−/−^ mice along with increased ChREBP binding to the *L-pk* promoter in these mice. It is thus possible that LXRα, LXRβ, and ChREBPα collectively integrate different sugar metabolites with lipogenesis in a wild type context. Acetyl-CoA generated by the ACL enzyme, which was strongly upregulated by fructose feeding (Figure 1B), also acts as a substrate for protein acetylation [49]. Metabolic sensing by LXRs and ChREBP involves modification by *O*-GlcNAcylation and acetylation [50,51] and there seems to be interplay between OGT and the acetylating p300 transcriptional coregulator [52], which is likely to impact LXR and ChREBP activity [53].

We did not observe reduced levels of nuclear *O*-GlcNAcylated proteins when knocking out LXRα, suggesting that both LXRα and β must be depleted for this phenotype. Because *O*-GlcNAc modification of ChREBPα is important for its activity and because ChREBPα was more modified in fructose fed LXRα^−/−^ mice, it would be interesting to study if the dietary effects of fructose on DNL gene expression and ChREBPα activity would have been more pronounced in an animal model where both LXR isoforms were depleted.

In summary, we provide evidence that hepatic expression of *de novo* lipogenic genes require LXRα activity in response to dietary glucose and that LXRβ may compensate for the lack of LXRα in fructose fed mice. As aberrant *O*-GlcNAc signaling during nutrient stress and diabetes leads to excessive glucose production and lipid accumulation in the liver [54], studies including mutated O-GlcNAc residues in LXRs and ChREBPα will provide a better understanding of the relevance of coordinated *O*-GlcNAcylation of these transcriptional regulators under physiological and pathophysiological conditions.

## Figures and Tables

**Table 1:** Body weight, food intake, and plasma insulin of fasted and refed mice.

Genotype	Treatment	Body Weight (g)	Food Intake (g)	Plasma Insulin (μg/dL)
LXRα^+/+^	Fasted	30.8 ± 2.0		0.7 ± 0.1
	Refed-Fructose	28.4 ± 1.7	2.4 ± 0.4	2.0 ± 0.7
	Refed-Glucose	29.6 ± 2.3	3.3 ± 0.2	7.0 ± 1.0 ***
LXRα^−/−^	Fasted	32.7 ± 1.5		0.7 ± 0.1
	Refed-Fructose	31.1 ± 2.9	2.8 ± 0.2	5.0 ± 1.5 *
	Refed-Glucose	32.1 ± 3.5	2.7 ± 0.4	7.1 ± 0.8 ***

Wild type (LXRα^+/+^) and LXRα knockout (LXRα^−/−^) mice were treated as explained in Materials and Methods. Values are mean ± standard error of the mean (SEM) (*n* = 5). Significant differences were calculated by two-way analysis of variance (ANOVA) followed by Tukey’s multiple comparisons test. * *p* < 0.05, *** *p* < 0.001 compared to fasted within same genotype.

## Supplementary Materials

The following are available online at www.mdpi.com/2072-6643/9/7/678/s1. Figure S1: Quantification of cytosolic and nuclear proteins LXRα/β, SREBP-1 and ChREBPα. Western blot quantification of cytosolic (A) and nuclear (B) proteins was analyzed by ImageJ. Data represent the mean ± SEM (*n* = 3–4). Significant differences were found using two-way ANOVA followed by Tukey’s multiple comparison test. ** *p* < 0.01, *** *p* < 0.001 compared to fasted. ^#^ *p* < 0.05, ^##^ *p* < 0.01, ^###^ *p* < 0.001 compared to LXRα^+/+^ mice. Figure S2: Quantification of nuclear lysates input and WGA enriched proteins LXRα/β, ChREBPα and *O*-GlcNAcylation levels (RL2). Western blot quantification of cytosolic lysates (A), nuclear lysates input (B), and WGA enriched (C) proteins were analyzed by ImageJ. Data represent the mean ± SEM (*n* = 4). Significant differences were found using two-way ANOVA followed by Tukey’s multiple comparison test. * *p* < 0.05 compared to fasted. ^#^ *p* < 0.05, ^##^ *p* < 0.01, ^###^ *p* < 0.001 compared to LXRα^+/+^ mice. Table S1: Mouse SYBR Green Primers sequences. Table S2: Metabolomics analysis of liver tissues of wild type and LXRα^−/−^ mice.
