# Supplementary material for: LXRα Regulates Hepatic ChREBPα Activity and Lipogenesis upon Glucose, but Not Fructose Feeding in Mice
Source: Nutrients. 2017 Jun 29;9(7):678. doi: 10.3390/nu9070678 (PMC5537793; doi:10.3390/nu9070678)

Supplemental Figure S1.

A. Cytosol

B. Nucleus

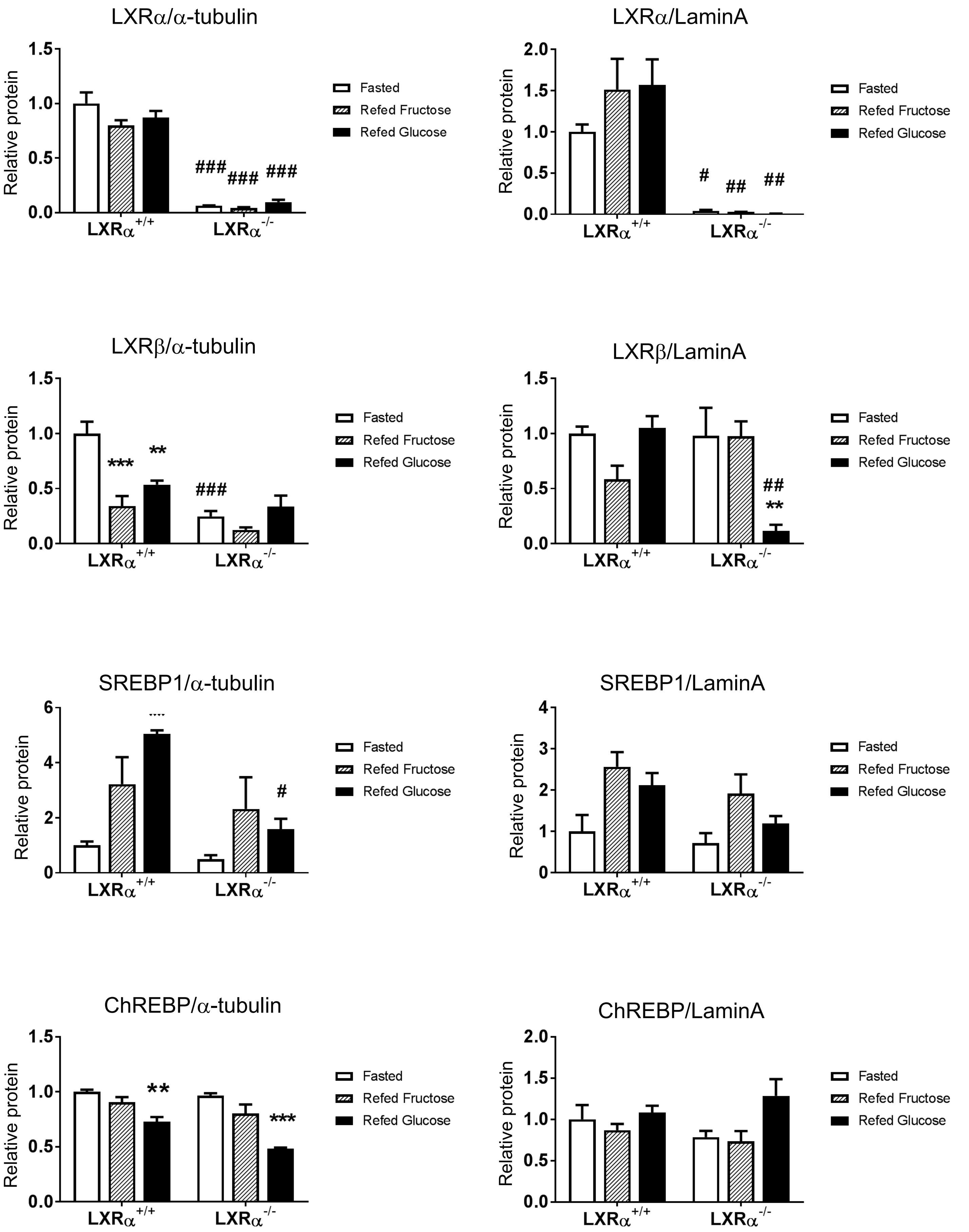

Supplemental Figure S2.

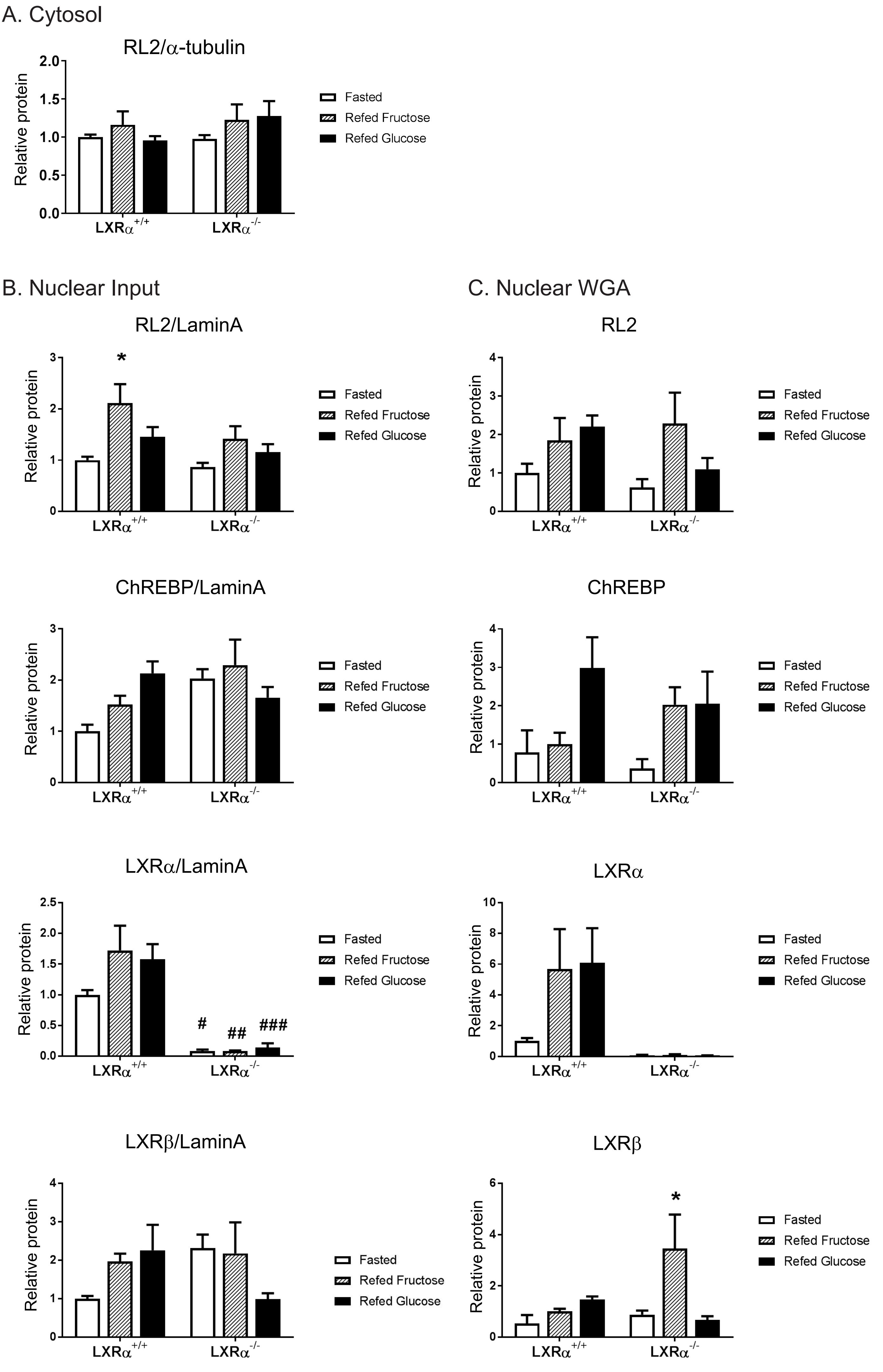

Supplement: Supplementary file 1 [file nutrients-09-00678-s001.zip › nutrients-203509-Suppl Figure.pdf]
