# Supplementary material for: LXRα Regulates Hepatic ChREBPα Activity and Lipogenesis upon Glucose, but Not Fructose Feeding in Mice
Source: Nutrients. 2017 Jun 29;9(7):678. doi: 10.3390/nu9070678 (PMC5537793; doi:10.3390/nu9070678)
Supplement: Supplementary file 1 [file nutrients-09-00678-s001.zip › nutrients-203509-Suppl Table S1.pdf]

Supplementary Table S1. Mouse SYBR Green Primers sequences.

Gene Name	Gene Symbol	Abbreviation in Text and Figures	Primer Forward	Primer Reverse
Acetyl-CoA carboxylase-α	Accα	ACCα	TGTACAAGCAGTGTGGGCTGGCT	CCACATGGCCTGGCTTGGAGGG
Acetyl-CoA carboxylase-β	Accβ	ACCβ	TCCTTCCAGAACTCCTCCCG	GACATGCTGGGCCTCATAGT
ATP citrate lyase	Acly	ACL	GCCCCAAGATTCAGTCCCAAG	GCCTTGGTATGTCGGCTGAA
Aldolase B	Aldob	AldoB	AAGCGGGCTATGGCTAACTG	CTGTGAAGAGCGACTGGGTG
MLX interacting protein-like	Mlxipl	ChREBPα	CGACACTCACCCACCTCTTC	TTGTTCAGCCGGATCTTGTC
		ChREBPβ	TCTGCAGATCGCGTGGAG	CTTGTCCCGGCATAGCAAC
Fatty acid synthase	Fasn	FASN	AGATGGAAGGCTGGGCTCTA	GGCGTCGAACTTGGAGAGAT
Ketohexokinase (Fruktokinase)	Khk	FK	AACTCCTGCACTGTCCTTTCCTT	CCACCAGGAAGTCGGCAA
Glucose-6-phosphatase, catalytic	G6pc	G6Pase	GCTGGAGTCTTGTCAGGCAT	CGGAGGCTGGCATTGTAGAT
Glucokinase	Gk	GK	AACGACCCCTGCTTATCCTC	CTGCTCTACCAGAGTCAACGAC
Solute carrier family 2 (facilitated glucose transporter), member 2	Slc2a2	Glut2	ATCGCTCCAACCACACTCAG	GCTGAGGCCAGCAATCTGAC
Pyruvate kinase liver and red blood cell	Pklr	L-PK	CAGCAGTATGGAAGGGCCAG	AGTTGCTGCTGCTGGAAGAA
Nuclear receptor subfamily 1, group H, member 3	Nr1h3	LXRα	GGAGTGTCGACTTCGCAAATG	CAGCACACACTCCTCCCTCA
Nuclear receptor subfamily 1, group H, member 2	Nr1h2	LXRβ	GCTCTGCCTACATCGTGGTCA	TGCGCTCAGGCTCATCCT
Phosphoenolpyruvate carboxykinase 1, cytosolic	Pck1	PEPCK	CCTAGTGCCTGTGGGAAGAC	AAGTTGCCTTGGGCATCAAAC
Stearoyl-CoA desaturase 1	Scd1	SCD1	AAAGCCGAGAAGCTGGTGAT	TACAAAAGTCTCGCCCCAGC
Sterol regulatory element binding transcription factor 1	Srebf1	SREBP-1c	GGAGCCATGGATTGCACATT	GCTTCCAGAGAGGAGGCCAG
TATA box binding protein	Tbp	TBP	GCACAGGAGCCAAGAGTGAA	TAGCTGGGAAGCCCAACTTC
